# A Novel Deep Neural Network Method for HAR-Based Team Training Using Body-Worn Inertial Sensors

**DOI:** 10.3390/s22218507

**Published:** 2022-11-04

**Authors:** Yun-Chieh Fan, Yu-Hsuan Tseng, Chih-Yu Wen

**Affiliations:** 1 Simulator Systems Section, Aeronautical System Research Division, National Chung-Shan Institute of Science and Technology, Taichung 407, Taiwan; 2 Department of Electrical Engineering, National Chung Hsing University, Taichung 402, Taiwan; 3 Department of Computer Science and Engineering, National Chung Hsing University, Taichung 402, Taiwan; 4 Innovation and Development Center of Sustainable Agriculture (IDCSA), National Chung Hsing University, Taichung 402, Taiwan

**Keywords:** human activity recognition, variational autoencoder, generative adversarial networks

## Abstract

Human activity recognition (HAR) became a challenging issue in recent years. In this paper, we propose a novel approach to tackle indistinguishable activity recognition based on human wearable sensors. Generally speaking, vision-based solutions struggle with low illumination environments and partial occlusion problems. In contrast, wearable inertial sensors can tackle this problem and avoid revealing personal privacy. We address the issue by building a multistage deep neural network framework that interprets accelerometer, gyroscope, and magnetometer data that provide useful information of human activities. Initially, the stage of variational autoencoders (VAE) can extract the crucial information from raw data of inertial measurement units (IMUs). Furthermore, the stage of generative adversarial networks (GANs) can generate more realistic human activities. Finally, the transfer learning method is applied to enhance the performance of the target domain, which builds a robust and effective model to recognize human activities.

## 1. Introduction

Body area network (BAN) [1] is a wireless network consisting of inertial measurement units (IMUs) that are placed over the body. The function of a BAN is to track the body movement and gestures using multiple sensors. The on-body inertial sensors are widely used in the domain of human activity recognition such as video gaming, medical application, image graphics, and skill training. In healthcare, sensor data can be utilized to monitor the daily activities of patients and detect sudden events such as falling down. In the military, due to the limited physical space and training facilities, body tracking can be utilized to train soldiers immersed in the battle field scenarios such as virtual training simulators, which can capture body movements such as walking, running, shooting, and crouching in a virtual environment. Considering the difficulty of tracking and recognizing locomotive motions from soldiers by using a single camera, the authors [2,3,4] exploit Microsoft Kinect to capture color and depth data from the human body. However, to recognize different human actions by tracking the front view of the human skeleton is a challenging issue. Therefore, the above methods may be not feasible in a real-time virtual environment for military training.

In addition, on-body sensors have merits regarding privacy concerns and do not reveal more personal privacy than cameras. Furthermore, inertial sensors can be worn anywhere regardless of fixed-cameras location for image recognition. Moreover, RGB cameras usually work in a sufficient lighting-source room. Therefore, in order to develop a training simulator that is capable of capturing soldiers’ accurate postures and movements, a BAN is the proper bridge to connect the physical and virtual environments. In addition, a human activity recognition system can be built by wearable sensors on the human body, due to the advancements in lowering the energy consumption and increasing the computational power of inertial sensors.

In our previous work [5], a motion tracking system is proposed to monitor posture and gesture interactions between soldiers and the virtual environment via an inertial BAN method. The wireless BAN deployed over the full body of a soldier is composed of accelerometers, gyroscopes, and magnetometers. A kinematic chain is built to describe the relationship between rigid body movements and motions of joints. However, the main problem of wearable sensors is drifting in the wild. The wearable sensors deployed on the body keep changing positions and suffer from signal interference when the user is moving or performing activities. Moreover, the complementary filter in this motion-tracking method is ineffective to recognize a variety of rapid or complex actions. Paulich [6] presents evidence that the best error performance of a single wireless inertial sensor ranges from $0.51^{{}^{\circ}}\;$ to $1.65^{{}^{\circ}}\;$ RMS during human daily activity. In contrast, Marcard [7] indicates that a set of IMUs deployed over the body achieves only $16.9^{{}^{\circ}\;}$ average geodesic distance between ground-truth and IMUs, compared with a better result of $12.1^{{}^{\circ}}\;$ using vision and inertial fusion solution.

To address the above issues, we propose a multistage deep neural network (DNN), which is less dependent on the handcrafts feature extraction, comparing to other famous machine learning algorithms, such as support vector machine (SVM), naive Bayes, and random forest (RF) models. Since the amount of labelled data is not sufficient to train the DNN model on indistinguishable human activities, we leverage the issue and propose a novel approach to train unlabeled data, integrating sensor fusion, an autoencoder, a generative adversarial network (GAN), and a convolutional neural network (CNN) classifier. An autoencoder is mainly utilized for exploratory data analysis to discover patterns among data, is used in the pre-training process, and is efficient on feature extraction and dimensionality reduction. The key point for applying a GAN is to generate synthetic data for HAR and we adopt the transfer learning method to recognize activities of team members. We retrained the team modal from a well-trained GAN model via the limited amount of team training data. In addition, a dense layer is added on the top of classifier layers and the model parameters of the collective sensor data of several trainees are reused to retrain the team activity for advanced recognition of team activities. The transfer learning method helps enhance the performance of specific activity recognition.

Therefore, with the customized deep neural network method, the classification accuracy of the real dataset can be boosted. The proposed method helps avoid mismatched actions from the former single trainee activity classifier, which corrects the team training problems. The presented approach can achieve accuracy improvement compared to other methods [8,9,10,11,12]. The major contributions and key features of this paper are:

- Proposing a human activity recognition approach based on DNN for classification with a GAN model;
- Presenting a variational autoencoder (VAE) model method to denoise and extract the signal from inertial sensors in the environment;
- Using synthetic skeleton information to recognize the activities of individuals without using a vision approach;
- The proposed system can be scaled up to a ten-sensor deployment of the whole body or scaled down to two-sensor deployment of the human body;
- Recognizing the activities of a group or a team via pre-training model-based transfer learning. When it comes to the training dataset, the proposed method is limited due to insufficient activity data for training the neural network, especially when the transfer learning method cannot play a role in improving the accuracy of team activity recognition. With the help of a transfer learning approach, the model is simply retrained with the target data, conducted with fewer training epochs. Therefore, with scarce data, the proposed system can be utilized to achieve better classification accuracy.

The organization of this paper is as follows: Section 2 reviews related works about inertial-sensor-based HAR approaches. Section 3 presents the proposed system architecture. Section 4 evaluates the system performance and presents the performance comparison. Finally, Section 5 draws conclusions and shows future research directions.

## 2. Related Works

Inertial sensors have been utilized for human activity recognition due to several advantages, such as being wearable, light weight, and the ability to use plenty of sensors, where the IMUs can collect activity data over a period of time (e.g., detecting emergency events or recognizing daily activities). To classify different actions or activities, several machine learning approaches are proposed for feature extraction. For instance, k-nearest neighbor (kNN), SVM, RF [13], hidden Markov model (HMM) [14,15], and decision tree (DT) models. The authors [16,17,18] use machine learning such as kNN and SVM to detect the patient falling events. The authors of [19] and [20] present that the methods using SVMs are capable of improving the recognition accuracies. Considering the HAR as a general machine learning problem that requires feature extraction and feature selection, Suto et al. [21,22] emphasize the importance of feature selection on HAR problems, and further investigate the performance of feature selection methods in combination with machine learning algorithms, such as feed-forward artificial neural network, kNN, and decision tree. Notice that these methods have a high classification rate with respect to a few daily activities, and hand-crafted features are the key points for increasing their accuracies.

In recent years, artificial neural networks have shown a different impact on human activity recognition. The multilayer perceptron with multilayer feed-forward architecture can deal with complex activity classification for the ability of non-linear activation. DNN methods for action recognition were proposed in recent years [23,24]. Moreover, a CNN model can benefit the body skeleton and extract the information from the characterization of the skeleton of human, e.g., [25,26,27]. The authors in [26] use the CNN approach to acquire time-series data and build a multilayer classifier for sequences of skeleton recognition.

Recurrent neural networks (RNNs) are also an approach to capture the spatial temporal evolution of the skeleton. The existing methods adopted gated RNN architectures such as the long short-term memory network (LSTM) [28,29]. The authors use the LSTM method to combine the video and the inertial sensors for pose prediction [30]. The work illustrates how a complex RNN model copes with the inertial sensors and a sequence of images for action classification [31]. The interested reader may refer to [32,33] for further discussion of action recognition using data sensing techniques and deep learning methods.

One of the crucial factors of raising the accuracy of human activity recognition rate is the sufficient amount of training data. However, acquiring sufficient data is difficult under this circumstance. Thus, a GAN is developed to learn a hidden structure of the data from the original distribution and then generate new data within the same distribution. Several GAN models [34,35,36,37,38,39] are proposed to generate verisimilar sensor data for recognizing the actions, where the generated data allow the use of data augmentation in HAR to improve the accuracy. The comparison of the state-of-the-art methods using sensory data with the GAN model are listed in Table 1.

To characterize the diversity with a multi-dimensional dataset, smart metasurfaces, platforms for multiple sensors, are proposed. As described in [43], the smart metasurface is capable of sensing ambient environments by integrating an additional sensor(s) and can adaptively adjust its electromagnetic operational functionality through an unmanned sensing feedback system. A motion-sensitive smart metasurface integrated with a three-axis gyroscope is developed to illustrate the feasibility of the metasurface platform. Moreover, based on a multi-layer digital-coding metasurface array, Liu et al. [44] report a programmable diffractive deep neural network for handling various applications such as wireless communications, signal enhancement, medical imaging, remote control, and the Internet of Things, which may further extend the applications of the proposed algorithm framework.

## 3. System Description

**Figure 1:**
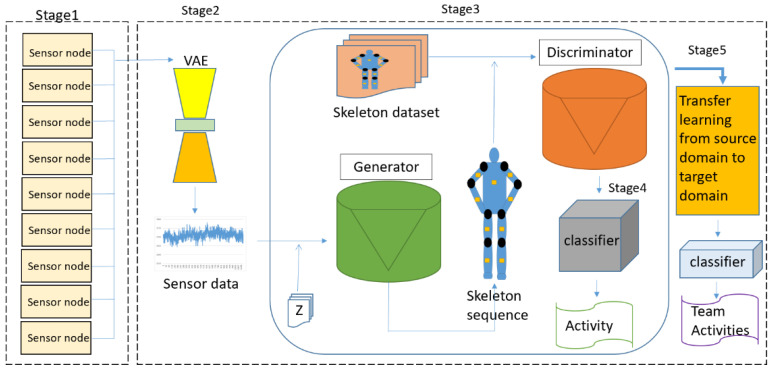
The proposed DNN architecture on HAR.

As mentioned above, human action recognition is crucial to develop an immersive training simulator. The wireless BAN can immediately track the action of the skeleton of the trainee. The system operations consist of four stages: (1) sensor fusion, (2) a VAE, (3) a GAN, and (4) CNN-based classification. We use a VAE to pre-train the sensing data and extract crucial features. A VAE encodes the nine-axis data into a feature-representation vector, constrained by Gaussian distributions. The VAE stage aims to denoise the sensing measurements, which transforms into a family of Gaussian distributions in the latent space with mean and variance. Our goal is to build 2D skeleton maps from the inertial sensors and to recognize the activities from the skeleton maps. To deal with the data from the VAE process, the deep convolutional GAN (DCGAN), which consists of two major modules, a skeleton map generation module and an activities recognition module, is applied. Figure 1 shows the proposed DNN architecture for human activity recognition.

### 3.1. Stage 1: Sensors Fusion

**Figure 2:**
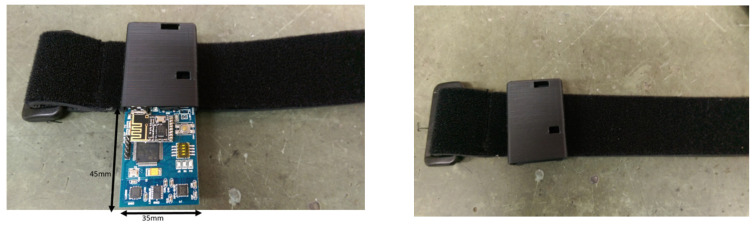
Top view of sensor nodes (**left**); the wearable sensor node (**right**).

**Figure 3:**
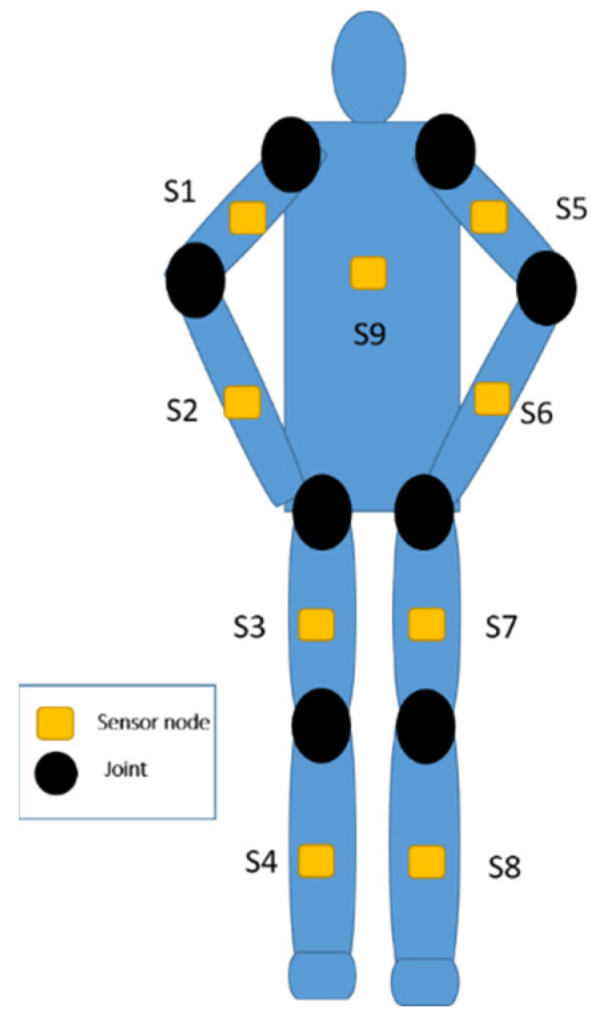
The deployment of inertial sensors over the body.

This section introduces the sensors used in the BAN. The sensor nodes are nine axial inertial measurement units (IMUs), which are equipped with an ARM Cortex-M4 microprocessor and ST-MEMS chips. Figure 2 illustrates the appearance of the sensor hardware. The customized sensor can capture three-axis acceleration, three-axis angular velocity, and three-axis magnetic strength, which transmits data streaming via 2.4 GHz RF radio. The trainees wear multiple sensors on the arms, wrists, thighs, lower legs, and back. As depicted in Figure 3, the inertial signals are acquired from accelerometers, gyroscopes, and magnetometers for the T-pose action. The orientation of the sensors that are attached to human bones are measured in local frames employed in quaternion representation.

(1) $$q_{t_{1}}^{node}=q_{\Delta t}^{node}\;⊗q_{t_{0}}^{node},$$

where ⊗ is a quaternion multiplication operator, $q_{t_{0}}^{node}$ is defined as a sensor node’s quaternion output while the wearable sensor nodes is in the pose at time *t*_0_, and $q_{t_{1}}^{node}$ depicts a valid rotation after a time interval Δ*t*. Note that *t*_1_ = *t*_0_ *+* Δ*t* and $q_{\Delta t}^{node}$ is the rotation used for the orientation of the sensor node.

### 3.2. Stage 2: Variational Autoencoder (VAE)

**Figure 4:**
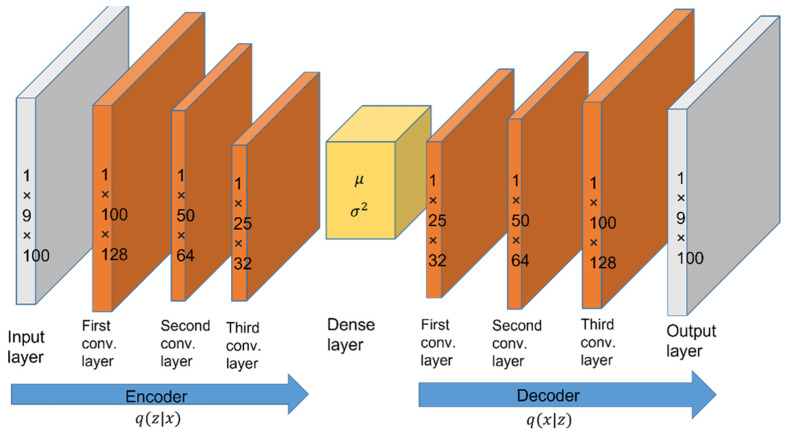
The proposed VAE architecture for data pre-training.

A VAE [45,46] can preserve the source information from sensor nodes, including three-axis acceleration, three-axis angular velocity, and three-axis magnetic strength data. The VAE aims to handle the sensor data from each IMU and transform better information from the orientation of sensors with measured noise. A sequence of sensors M = [s1, s2, ..., s9] is given from upper stage and becomes the input of the VAE, which consists of an encoder and a decoder. Figure 4 depicts the proposed VAE architecture, which contains both a CNN-based encoder part and decoder part for the data pre-training process. Both of them have three convolutional layers. The multiple layers of the VAE denoise data and extract the source information. In the encoding process, the input data are transformed into features that can reconstruct valuable inputs from the source. The decoding process is conducted in a similar way to perform influences after the three convolutional layers. We used 100 batches to train the VAE model and the Adam optimizer to minimize the loss with a learning rate of 0.01. The VAE reconstructed the sensing data as a less noisy signal and preserved the crucial information of inertial sensors. To extract the useful information from IMU sensors, three convolutional hidden layers and three deconvolutional layers were used for the encoder and the decoder. The loss function is defined as follows:

(2) $$L\left(x\right)=E_{q(z|x)}\log p(x\;|\;z)-D_{kl}[q\left(z|x\right)||p\left(z\right)],\;$$

where *q*(*z*|*x*) is the output of the convolutional encoder part, $D_{kl}$ is the Kullback–Leibler distance between *p* and *q*, and *p*(*z*) is the prior information for the latent variable and unit-variance Gaussian,

(3) $$p\left(x|z\right)=\frac{1}{\sigma \sqrt{2\pi }}e^{\frac{-{\left(x-f\left(z\right)\right)}^{2}}{2\sigma^{2}}},$$

where $\sigma^{2}$ is the variance and *p*(*x*|*z*) is the output of the convolutional decoder.

### 3.3. Stage 3: Generative Adversarial Network (GAN)

**Figure 5:**
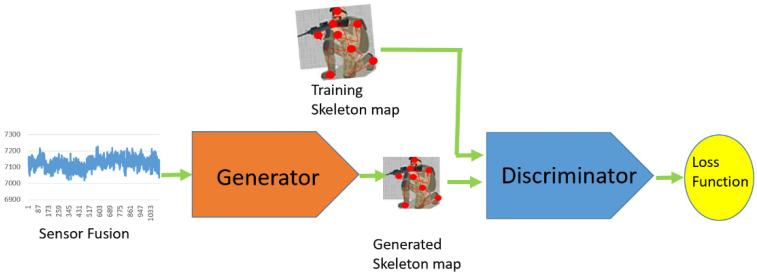
The proposed generative adversarial network.

Referring to the operations of the first two stages, a GAN is capable of producing a sequence of synthetic skeletons, consisting of a generator that attempts to capture the distribution and a discriminator that assesses the generator output and penalizes unrealistic samples. It is a network architecture that is trained to achieve an equilibrium. We receive sensor data from VAEs and then feed the data into GANs in order to generate entirely new and realistic skeleton maps that match the distribution of a given target dataset. The architecture for the proposed GAN model is illustrated in Figure 5. The generator yields a sequence of skeleton as the input of the discriminator. Uniquely, our approach utilizes a conditional GAN to generate a sequence of verisimilar skeletons from individuals on different activities. A small amount of sensor data of individuals are trained as labeled data with respect to the human activities. Given multiple sensor data of an individual, the generator tries to create a corresponding skeleton map for data augmentation. We train a sequence of the skeletons so that inferred skeletons are similar to real ones. Thus, the skeleton map derived from the generator is applied to distinguish the human activities by a discriminator.

**Figure 6:**
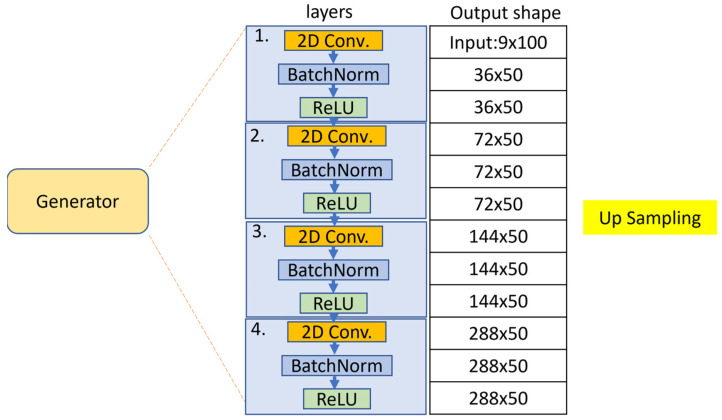
The block diagram of the generator.

**Figure 7:**
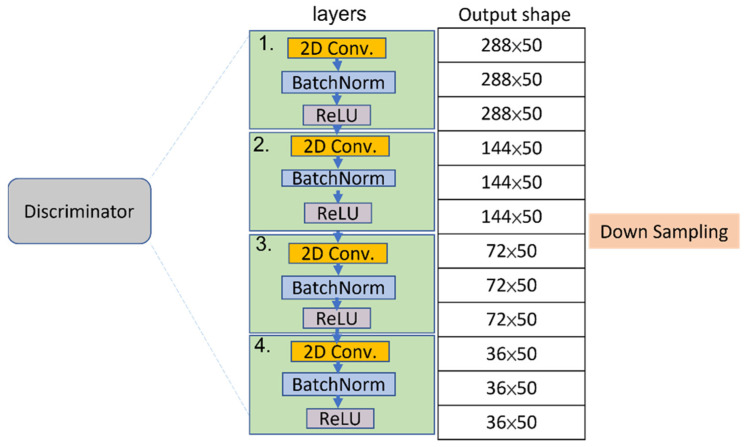
The block diagram of the discriminator.

The two modules, a sequence of skeleton generator and an activities discriminator, are trained by using the G and D loss functions, respectively. The GAN training is a strategy to define a game between the generator network and the discriminator network. The block diagrams of the generator and discriminator are illustrated in Figure 6 and Figure 7.

(4) L=VDmaxD,G= Gmin DmaxEx~Pdataxlog Dx+Ez~Pzzlog1−Dx^,

where *L* is the sum of GAN stage loss, *V* is the value of this minimax game strategy, *z* is a vector of random values, $P_{data}$ is the prior probability distribution of the real data, $P_{z}$ is the prior probability distribution of random input vectors *z*, and *x* is a sequence of the input VAE data. Since a GAN model is difficult to train and optimize from the generator’s output rather than the discriminator’s, a Wasserstein GAN (WGAN) [47] is used for IMUs data prediction. In the WGAN, we now utilize a gradient penalty to optimize the generator process.

(5) $$L_{g}=L_{gan}+\alpha L_{p},\;$$

(6) $$L_{gan\;}=-D\left(\hat{x}\right)=G\left(z\right),\;$$

(7) $$L_{p}={\left(||\nabla_{\hat{x}}D\left(\hat{x}\right)|{|}_{2}-1\right)}^{2}$$

where $L_{g}\;$ is the generator loss function, $L_{gan}$ is the original WGAN critic loss, and $L_{p}$ is the gradient penalty loss, where the operator || indicates concatenation.

### 3.4. Stage 4: CNN Classifier

**Figure 8:**
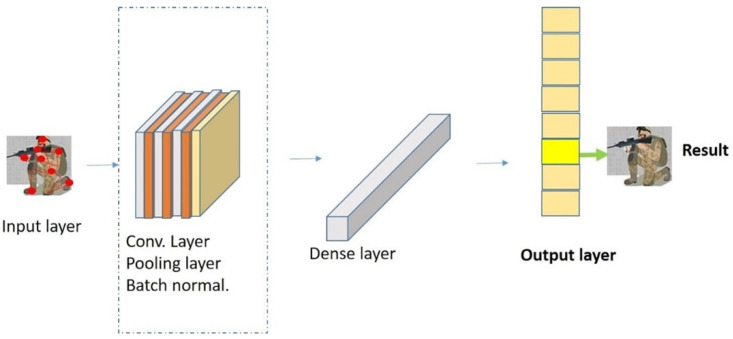
The design of the CNN classifier.

A CNN architecture is utilized to perform the classification of skeleton sequences derived from the output of Stage 3. The design of the CNN classifier is illustrated in Figure 8. Observe that the CNN consists of three convolutional layers, including a batch normalization layer, a max pooling layer, and a dense layer. Afterwards, the classification is performed via a SoftMax function, which yields:

(8) $$\mathrm{Softmax}\;\mathrm{classifier}=P\left(c|p\right)=\frac{\exp\left(p^{L-1}w_{c}^{L}+b_{c}^{L}\right)}{\sum_{k=1}^{Nc}\left(p^{L-1}w_{k}\right)},$$

where *c* is the activities class, *L* is the last layer index, $N_{c}$ is the total number of activities class, w is the weight, and b is the bias. We pre-train the CNN classification using augment data generated from GANs and fine-tune the model by using the real data.

### 3.5. Stage 5: Transfer Learning

Since collecting a sufficient amount of team training data may be difficult, there is a need for transfer learning methods from a well-trained source domain to the target team training domain. Hence, we can use the GAN model to train the little labeled data on mutual actions. The model with transfer learning is as efficient as the modal in the source domain. Moreover, a hidden layer is added on the top of the CNN classifier layers for classification [48,49]. The transfer learning method is applied to enhance the performance of the target domain, which is lacking a sufficient amount of the labeled data. We kept the optimizer, batch size, and learning rate the same as the previous model and retrained the current model with transfer learning, which prevented forgetting the previously learned knowledge for obtaining better classification accuracy in the target domain.

## 4. System Evaluation

In order to evaluate the proposed system, multiple experiments are conducted for human activities recognition on the public dataset: UCI OPPORTUNITY dataset. The software is Tensorflow Keras for Python. The computer hardware is equipped with 3.1 GHz Intel Core i7 processor, 8GB RAM, and a NVidia 2070 graphic card.

### 4.1. Data Feature

The UCI dataset is applied, which features four subjects who perform 17 different activities, such as opening a door, opening a drawer, and toggling a switch. The data are recorded every 5 min by using seven inertial sensors that are attached to the torso, right upper/lower arm, left upper/lower arm, right leg, and left leg. The sensor data of different activities recorded at a frame rate of 32 Hz were segmented into multiple 2 s windows.

### 4.2. Data Processing

**Figure 9:**
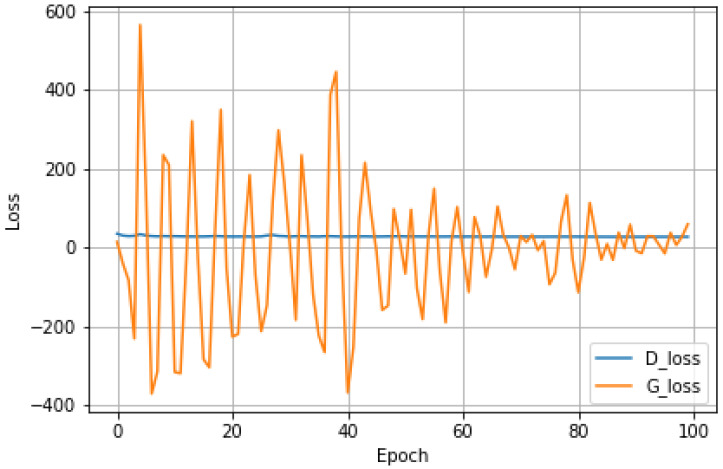
The discriminator and generator losses with a total of 100 epochs during the training process (blue: discriminator loss. Orange: generator loss).

The data from nine different measurements of the sensors (x, y, z gyroscopes, x, y, z accelerometers, x, y, z magnetometers) are preprocessed, normalized, and transferred to quaternion forms. Therefore, the pre-trained data are prepared for the variational autoencoder stage for training. The output of VAE is applied as the input for the GAN stage. After the GAN training, the generated synthetic output is processed via the final stage (i.e., a CNN classifier). The generator and discriminator are trained with the Adam optimizer with a learning rate of 1 × 10^−5^. A batch size of 100 is used for the GAN architecture, where the generator loss and discriminator loss converge to 59.92 and 27.68 after 100 training batches, respectively. The best generative loss is 4.41 for epoch 91. The result of generator and discriminator losses are depicted in Figure 9.

### 4.3. Assessing the Generated Data

We compare the generated data to several machine learning methods such as SVM, kNN, and decision tree. All parameters used in the machine learning methods are grid search. As shown in Table 2, the proposed method outperforms other machine learning methods in terms of average accuracies, which suggests that more information from the IMUs in our proposed method leads to better results, compared with other machine learning methods. There is an increment in the performance metrics for three different methods if the amount of IMUs increase, except in the decision tree method. The average accuracy of the SVM method increases from 90.6% to 92.9%, the kNN method from 89.1% to 90.6%, and the proposed method from 91.5% to 95.7%. Note that different activities are usually classified for upper or lower body movements. Hence, if we place fewer IMUs over the body, the accuracy is highly related to the sensor deployment strategy, which can obviously influence the accuracy of the result. Accordingly, in addition to using two wearable IMUs over the human body, we also present a multi-IMU deployment method that can be applied to clearly describe body movements. The DNN architecture is built to tackle specific HAR cases.

Moreover, to assess the impact of a random noise in the environment on system performance, we examine the performances of the proposed method, DNN method with CNN model, and LSTM model. As shown in Table 3, we summarize the quantitative results of mean accuracy and F1 score on sensory data with Gaussian noise (signal to noise ratio of 7.78 dB). In the training process, all the models were trained by feeding Gaussian noise to a sensor of the left leg in the experiment. Referring to the results, the proposed method outperforms other DNN models on HAR if there is a random noise in the environment. The mean accuracy reaches 88.4%, which is superior to the RNN (LSTM) model in [50] with 82.9% accuracy for the same selection of noisy sensor on test data of every subject.

### 4.4. Classification Performance

**Figure 10:**
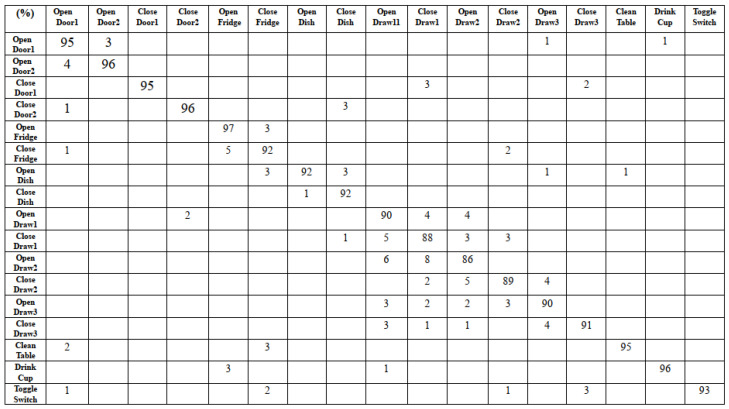
The confusion matrix result of our DNN method on different human activities.

To validate the classification performance, we evaluate the confusion matrix result on the UCI OPPORTUNITY dataset. As depicted in Figure 10, the accuracy of all activities trained by all sensors data ranges from 86% to 97%. Although the accuracy of open drawer, close drawer cases have lower classification results, the proposed method is able to benefit from the DNN model to improve the accuracy of most classification results. We also evaluate mutual action classification results.

The parameters are learned by the small amount of team training data. We combine two subjects with different actions (e.g., open a door, close a door) to be the target domain. The other actions can be the source domain. By using transfer learning method, we train the GAN model with target sensor data. Hence, the proposed model is able to recognize the different activities from small amounts of data. Accordingly, with the transfer learning method, the proposed method is trained on the UCI OPPORTUNITY dataset, which means the parameters of the hidden layer are renewed by a small amount of training data. Finally, the mean accuracy leads to an improvement of 12% compared with the proposed architecture, without introducing the transfer learning method.

**Figure 11:**
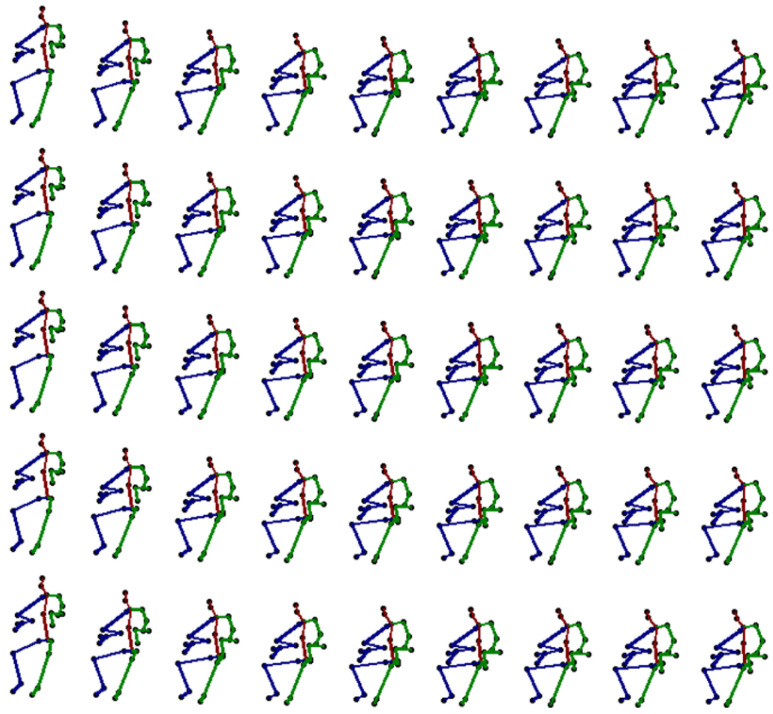
The synthetic sequences derived from the proposed DNN method, shown in 3D presentation (a sitting down action was performed by five different subjects).

Therefore, our solution raises the accuracy of motion recognition by building a DNN model on the measurement of sensors. The synthetic action sequences generated from the IMUs are deployed on five different subjects for two seconds. We depicted the data in a visual presentation. Figure 11 shows the sitting down action generated from the GAN model, which is able to enforce the motion sequence.

## 5. Conclusions

In this work, we propose a novel and supervised method for human activity recognition. The VAE and GAN models are able to improve the accuracy of action classification. Accordingly, we can recognize the motion of IMUs data even though the inertial signal is drifting over a long duration, which opens another door for our simulator to execute a correct motion or pose in the virtual reality operation. The on-body sensor deployment of the proposed system can be scaled up (e.g., a ten-sensor deployment) or scaled down (e.g., a two-sensor deployment). Compared to the existing deep, convolutional, and recurrent models in [50], the proposed method leads to significant improvement in HAR accuracy. When it comes to the training dataset, the proposed method is limited due to insufficient activity data for training the neural network, especially when the transfer learning method cannot play a role in improving the accuracy of team activity recognition, as described in Section 3.5 (page 8). Ideally, higher classification accuracy can be achieved with a more complete sensor dataset in the training process. In future work, we plan to collect more on-body HAR data and conduct efficient sensor deployment strategies so that the classification accuracy can be further improved.

## Figures and Tables

**Table 1:** The comparison of different sensory methods using for HAR with GAN model.

Methods	Purpose	IMUs	Advantages	Limitations
SensoryGAN [38]	Augmentation	Acc	The first work about data augmentation with synthesizing sensory data.	Using three activity-specific GANs for three activities.
Zhang [40]	Augmentation	Acc Gyro Mag	The first method that uses semi-supervised GAN for activity recognition.	Using some hand-crafted parameters to control the classification results.
Soleimani [41]	Augmentation	Acc	Generating data of the target domain from the source domain via GANs.	The diversity of the sensory data is limited.
Mathur [42]	Augmentation	Acc Gyro	Enriching the training set with sufficient discrepancy of different sensor deployments.	The diversity of the synthetic data is limited and not guaranteed.
Proposed Method	Augmentation	Acc Gyro Mag	Using synthetic sensory data to recognize the activities of individuals and a group.	Using limited team data to train the neural network.

**Table 2:** The accuracy of different methods on the different amount of IMUs.

	One IMU (%)	Two IMUs (%)	Five IMUs (%)	Average Result (%)
SVM	90.6	90.8	92.9	91.4
Decision tree	91.4	87.1	76.2	84.4
kNN	89.1	89.5	90.6	89.7
Proposed method	91.5	92.1	95.7	93.1

**Table 3:** The mean accuracy of different DNN methods on HAR.

	Model	CNN Model [50]	RNN (LSTM) Model [50]	Proposed Method
Metric				
Accuracy (without noise)		94.5%	95.4%	95.9%
F1 score (without noise)		93.7%	95.4%	95.8%
Accuracy (with noise)		78.5%	82.9%	88.4%
F1 score (with noise)		77.4%	81.7%	86.9%

## Data Availability

The author at the Simulator Systems Section, Aeronautical System Research Division, National Chung-Shan Institute of Science and Technology, Taiwan, was the subject of the experiments. The author consented to participate in this research study.
